# Arthroscopic triangular fibrocartilage complex reconstruction with free tendon graft for chronic distal radioulnar joint instability

**DOI:** 10.1186/s13018-021-02827-2

**Published:** 2021-11-17

**Authors:** Kuang-Ting Yeh, Jui-Tien Shih

**Affiliations:** 1 Department of Orthopedics, Hualien Tzu Chi Hospital, Buddhist Tzu Chi Medical Foundation, Hualien, 97002 Taiwan; 2 School of Medicine, Tzu Chi University, Hualien, 97004 Taiwan; 3 Department of Orthopaedic Surgery, Taoyuan Armed Forces General Hospital, NO. 168, Zhongxing Road, Longtan Dist, Taoyuan City, 32551 Taiwan

**Keywords:** Arthroscopy, Irreparable, Reconstruction, Triangular fibrocartilage complex, Wrist

## Abstract

**Background:**

Tears in the triangular fibrocartilage complex (TFCC) often manifest as ulnar wrist pain and limited wrist function. In chronic cases, the treatment of large tears with irreparable TFCC degeneration combined with distal radioulnar joint (DRUJ) instability is difficult. In the current report, we describe the outcomes of a minimally invasive technique for TFCC reconstruction using the free palmaris longus (PL) tendon via arthroscopy.

**Methods:**

We examined the cases of 67 adult patients [54 men and 13 women; age range, 19–34 years (mean age, 26.4 years)] treated for chronic and irreparable TFCC tears from 2001 to 2019. We used the arthroscopic TFCC reconstruction method with the free PL tendon for all chronic and irreparable TFCC injuries with DRUJ instability in our clinic. Thereafter, the patients underwent the rehabilitation program, which included wrist motion and occupational therapy. The mean time period from the event causing the tear to the operation was 22.6 months.

**Results:**

The function results of these patients significantly improved, and the ulnar wrist pain significantly decreased at postoperative follow-up. Of the 67 patients, 38 rated their wrists as “excellent,” 26 as “good,” and 3 as “fair.” None of the patients developed wound infections or complications.

**Conclusions:**

The results of this study suggest that arthroscopic TFCC reconstruction using the free PL tendon is an effective method for treating chronic and irreparable TFCC tears with DRUJ instability.

## Background

Tears in the triangular fibrocartilage complex (TFCC) are often indicated by ulnar wrist pain and limited wrist function. The TFCC facilitates the rotation of the radius around the ulna, which is the center of forearm rotation [1–3]. Thus, the TFCC is subjected to axial loads and shear forces. The TFCC is composed of the central fibrocartilage, the dorsal and palmar distal radioulnar ligaments, and the sheath of the extensor carpi ulnaris (ECU) tendon. The TFCC functions as a unit rather than as separate ligaments. The vascularity of the TFCC enables surgeons to repair acute peripheral tears, with excellent outcomes. However, in cases of chronic TFCC tears, the outcomes of TFCC repair are controversial. The TFCC performs three important biomechanical functions [4]. First, it enables the forearm to be loaded or stressed loading or stress passes from the forearm to the wrist via the TFCC. Second, the TFCC provides primary stability to the distal radioulnar joint (DRUJ). The TFCC is an important stabilizer of the DRUJ when the distal radioulnar ligaments become taut. Third, the TFCC stabilizes the ulnar carpus via the ulnar carpal ligament complex. If the TFCC is traumatized and disrupted, the wrist may become unstable. Thus, tears cause progressive DRUJ instability and subsequent degenerative changes to the TFCC, lunate, ulna, and triquetrum, as well as loss of wrist range of motion and grip strength. Chronic and irreparable tears, including the degeneration of the TFCC combined with DRUJ instability, are difficult to treat. Therefore, in the current study, we describe the outcomes of a TFCC reconstruction using the free palmaris longus (PL) tendon via arthroscopy.

## Methods

In the current study, we examined 67 patients (54 men and 13 woman) treated for chronic TFCC tears from September 2001 to August 2019. The ages of the patients ranged from 19 to 34 years, with a mean age of 26.4 years. All patients had experienced trauma such as axial loading and hyperextension injury. The time period from the event causing the tear to surgery ranged from 11 to 28 months, with a mean duration of 22.6 months. All the patients experienced ulnar wrist pain, reduced grip strength, limited performance ability, tenderness over the ballotable area of the ulna, passive ulnar deviation, and ulna loading-induced pain.

The results of the DRUJ stress test and the fovea compression test were positive in all the patients. Reviews of patient medical histories confirmed that all patients had been previously treated elsewhere with oral nonsteroidal anti-inflammatory drugs (NSAIDs) or herbal medicine. Radiography was performed for all patients to identify any bone fractures or ulnar variance and to assess the presence of degenerative changes. A true lateral radiograph can be used to identify the presence of DRUJ subluxation. All patients underwent a bilateral wrist computed tomography (CT) scan to assess the relationship between the distal radius and ulna, and to assess the presence of DRUJ instability according to the criteria by Mino et al. [5]. The ulnar head of a normal DRUJ should lie between two traced lines that define the dorsal and palmar borders of the radius. All the patients underwent preoperative wrist magnetic resonance (MRI) study for evaluation of TFCC and carpal interosseous ligament tear.

The Mayo modified wrist score, including pain, work status, range of motion, and grip strength, was used to assess wrist function. Moreover, a dynamometer was used to measure grip strength on the injured and contralateral side of the wrist. The results were reported as a percentage of the data on the contralateral side, for normalization. The repeated wrist CT scans were repeated for all patients at 12–24 months after surgery to reevaluate the relationship between the distal radius and ulna. The comparisons of preoperative and postoperative functional results were statistically analyzed with the Statistical Package for Social Sciences Statistics, version 23 (IBM SPSS Statistics, Armonk, NY).

## Surgical procedure

The surgery was performed with the patients under general anesthesia. An upper arm tourniquet was applied, and the pressure was set between 200 and 250 mmHg. Then, the wrist was elevated and distracted with a traction towel and finger traps applied over the index and middle fingers. The 3–4 portal and 6R (or 6U) portal were created. All patients underwent an arthroscopic wrist examination, and the lesions of the TFCC tears were identified and classified according to Palmer’s classification. We reconstructed TFCC with palmaris longus tendon graft under the indication as follows: 1. They had prominent ulnar wrist pain and unrecoverable wrist function more than 1 year after the history of trauma. 2. We found irreparable TFCC tear with concomitant Palmer’s type I (traumatic) and type II (degenerative) injury under arthroscopy. When the patients met the criteria, we performed PL tendon harvesting via the volar approach.

**Fig. 1:**
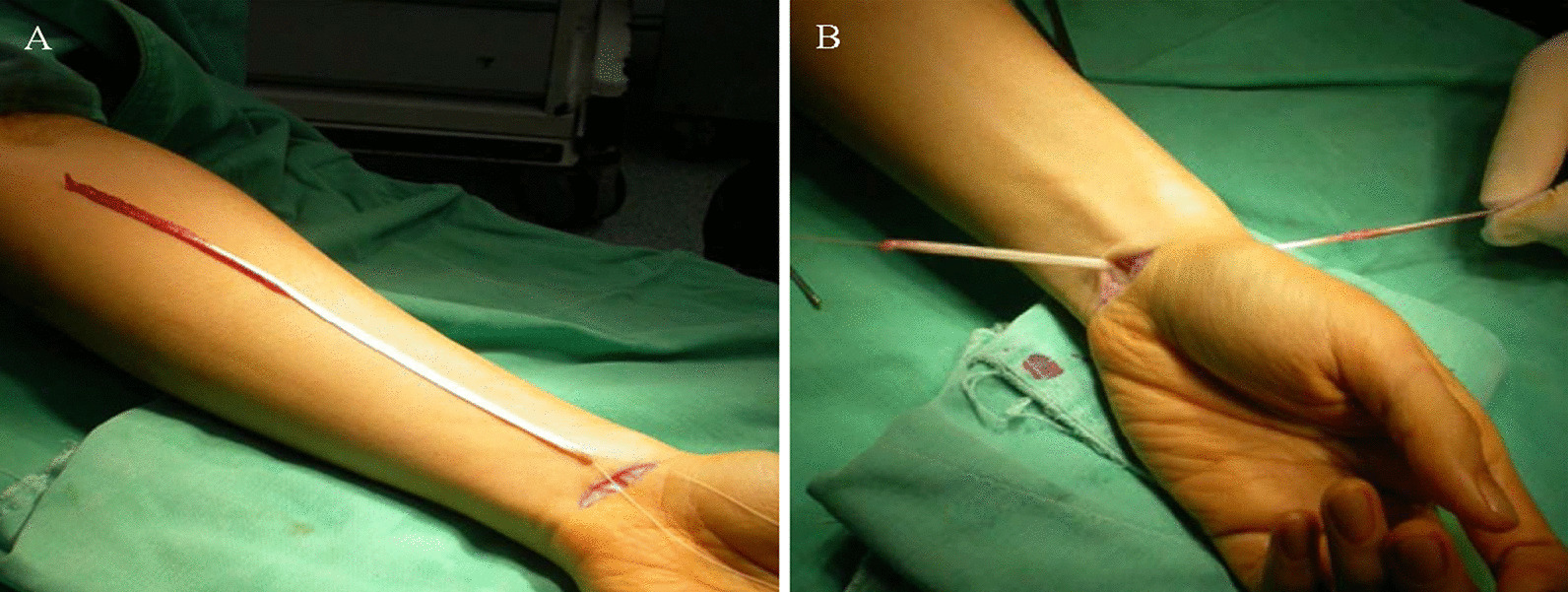
Palmaris longus tendon graft for reconstruction. **A** Palmar longus tendon is taken from the volar side of the wrist. **B** The radial tunnel was created just around the distal radioulnar joint of the distal radius, which permitted the tendon graft to pass from the volar to the dorsal side of the wrist

**Fig. 2:**
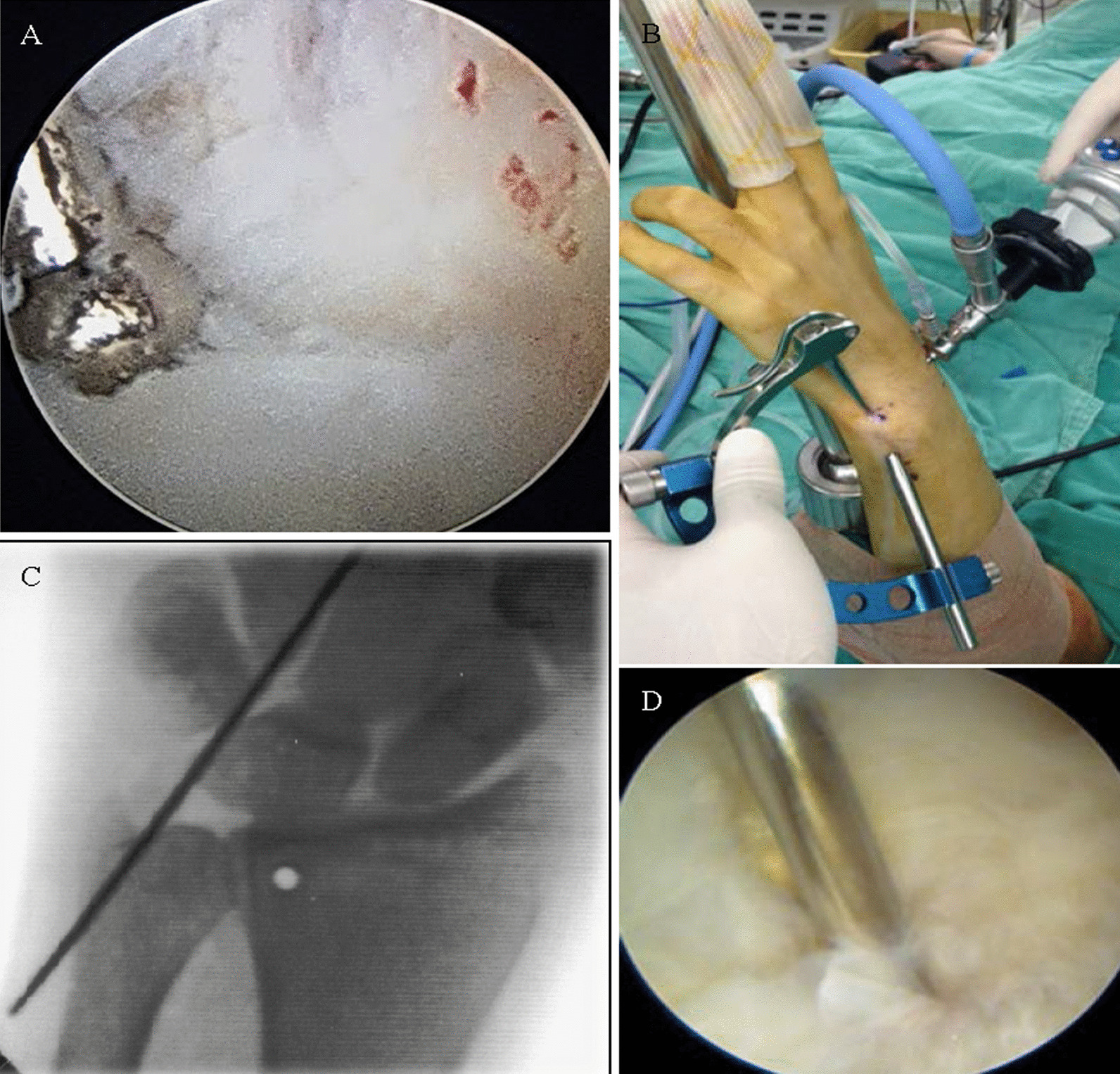
Fovea preparation and ulnar tunnel creation. **A** The fovea region of the distal ulna was identified under arthroscopy. **B** The microvector drill guide was used to create an ulnar tunnel for anatomic insertion of the tendon graft. **C** The direction of pin could be confirmed by the fluoroscopy. **D** The intraarticular exit of the tunnel was confirmed through the fovea under arthroscopy

The bony tunnel creation method was similar to Tse’s approach [6]. An incision was made approximately 1–2 cm over the palmar crease region. The PL tendon was identified and isolated (Fig. 1A). A 2 cm dorsal incision was made proximally from the 4/5 portal. The distal radius dorsal surface adjacent to sigmoid notch was exposed, and a 1.1 mm guide pin was inserted toward the volar surface as the radial tunnel. The exit point was around 5 mm from the sigmoid notch edge confirmed by fluoroscopy and out of the skin from the PL harvesting wound. Stepwise drilling of the radial tunnel to 2.5 mm for the passage of the PL tendon through the volar side to the dorsal side (Fig. 1B). The tears and inflammatory synovium were debrided using a small joint power shaver. The fovea region of the distal ulnar head was identified and prepared (Fig. 2A). Another 2 cm wound was made over 1 cm proximal to the tip of ulna styloid. A 1.1 mm guide pin was inserted through the microvector drill guide (Smith & Nephew Inc., Andover, MA, USA) targeting at the fovea of the ulnar head to create a bony tunnel was from the ulnar border to the fovea region of the distal ulnar head (Fig. 2B). The ulnar tunnel was then dilated to 2.5 mm width through stepwise drilling for the tendon graft smooth passage (Fig. 2C, D).

**Fig. 3:**
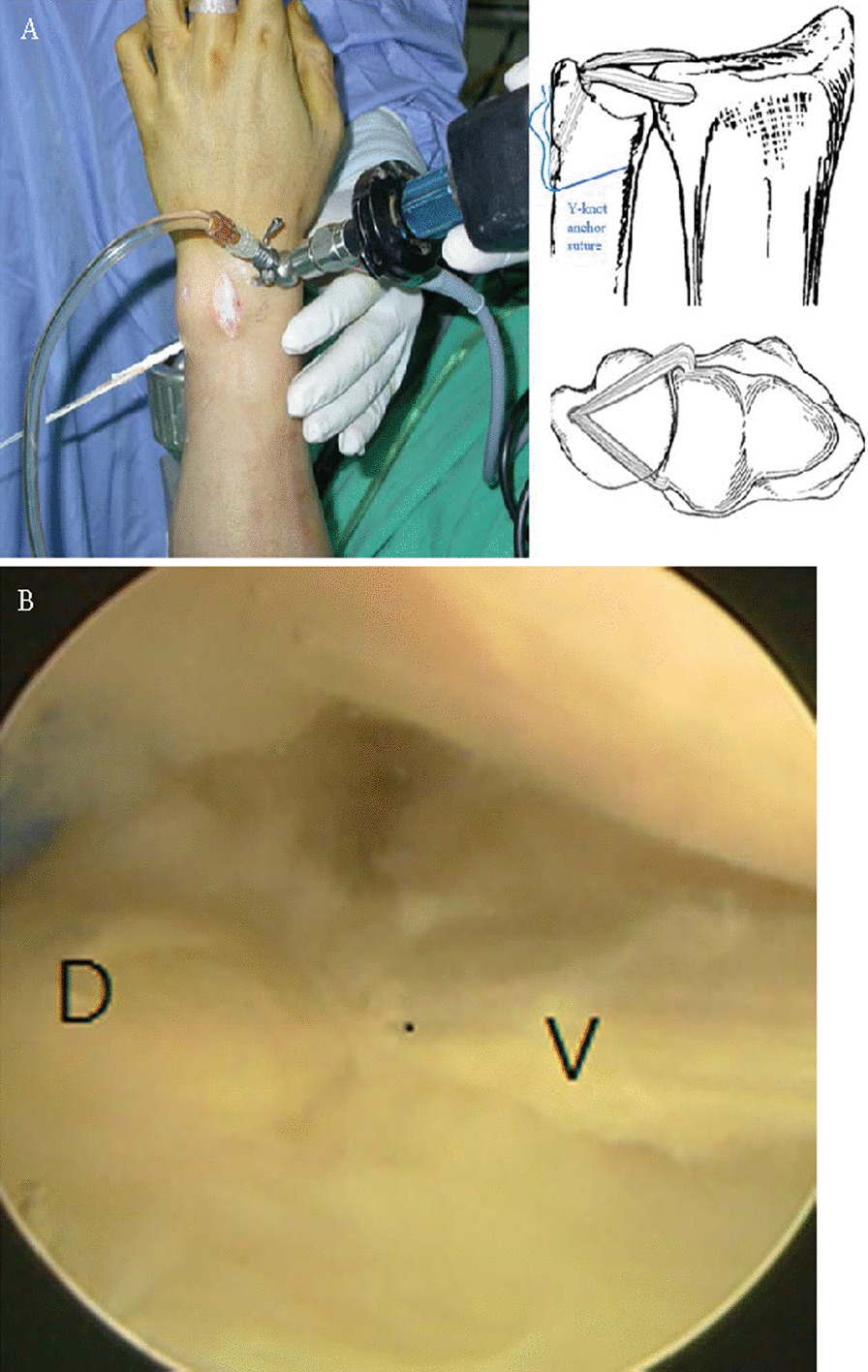
Anatomic triangular fibrocartilage complex reconstruction using free palmaris longus tendon and intraoperative image of pass of tendon graft into bony tunnel. **A** Both limbs of the graft were pushed back into the ulnar tunnel and the schematic diagram for both limbs position. **B** Arthroscopic confirmation of both limbs of the graft around the fovea region of the ulna. D = dorsal limb of the graft; V = volar limb of the graft

A trocar was introduced from the 4/5 portal and exited through the volar capsule for the insertion of the arthroscopic grasper approaching the volar limb of the PL graft into the joint. After retrieving the volar limb and the dorsal limb of the PL graft, respectively, into the ulnocarpal joint, we placed the suture passer through the ulnar tunnel to pull both limbs back into the ulna tunnel through the fovea, the ulnar tunnel and then outside the ulna (Fig. 3). A 1.3 mm Y-knot anchor suture [Conmed, New York, USA] was implanted adjacent to the ulnar tunnel extraarticular site. The graft was then sutured with the two sutures of the anchor for augmentation and then suture them together with surrounding tissue of the ulnar neck region (Fig. 3A). The condition of carpal interosseous ligament integrity was then routinely checked under arthroscopy of the midcarpal joint. The ulnar shortening procedure was then performed if the presence of a positive or zero ulnar variance preoperatively.

After surgery, a short arm splint was applied to the patient’s wrist for 4 weeks. A rehabilitation and strength exercise program were initiated 6 weeks after the operation. Pre- and postoperative wrist functions were evaluated using Mayo modified wrist scores before the operation and at 3 months after the operation, respectively. All surgeries were performed by a single surgeon, and patients were assessed and followed up at the outpatient department.

## Results

There were 54 male and 13 female included into this retrospective study with mean age as 26.4 years old (Table 1). There was no significant difference at preoperative wrist function and pain between male and female patients. The mean period of their symptom of ulnar wrist pain and dysfunction was 18.8 months (14–26 months). There were 37 (55.2%) patients who had negative TFCC findings at MRI study, but the pathology was confirmed through arthroscopy.

**Table 1:** Demographics

	Male	Female	Total	*p* Value
N	54	13	67	
Age	26.1 ± 4.3	27.7 ± 4.5	26.4 ± 4.4	0.246
Mayo modified wrist scores (Preop)	30.0 ± 2.7	29.6 ± 1.9	29.9 ± 2.6	0.646
Grip strength ratio (Preop)	37.1 ± 1.3	37.3 ± 1.6	37.2 ± 1.3	0.663
VAS of ulnar wrist pain (Preop)	6.3 ± 0.8	5.9 ± 0.9	6.2 ± 0.8	0.166

Data are presented as n or mean ± standard deviation. *p value < 0.05 was considered statistically significant after test

Fifty-two patients (77.6%) received ulnar shortening procedure during the operation. The ulnar variance in all the patients was all confirmed negative and was averagely 1.6 mm length. The mean postoperative follow-up duration of 32.2 months. None of the patients complained of moderate to severe wrist pain during their daily activities. Mayo modified wrist scores improved from 29.9 ± 2.6 preoperatively to 55.1 ± 1.0 postoperatively, while VAS of the ulnar wrist pain decreased from 6.2 ± 0.8 preoperatively to 1.6 ± 0.6 postoperatively (Table 2). Of the 67 patients, 38 (56.7%) achieved “excellent” results, 23 (34.3%) had “good” results, and 3 (4.5%) had “fair” results. Their grip strength ratio improved from 37.2 ± 1.3% preoperatively to 82.9% ± 4.8% postoperatively (Table 2). None of the patients developed surgical site infection.

**Table 2:** Score change before and after surgery (*n* = 67)

Item	Pre-OP	Post-OP	Diff	*p* Value
Mayo modified wrist scores	29.9 ± 2.6	55.1 ± 1.0	25.2 ± 3.0	< 0.001*
Grip strength ratio	37.2 ± 1.3	82.9 ± 4.8	45.7 ± 5.0	< 0.001*
VAS of ulnar wrist pain	6.2 ± 0.8	1.6 ± 0.6	− 4.6 ± 1.0	< 0.001*

Data are presented as n or mean ± standard deviation. *p value < 0.05 was considered statistically significant after test

**Fig. 4:**
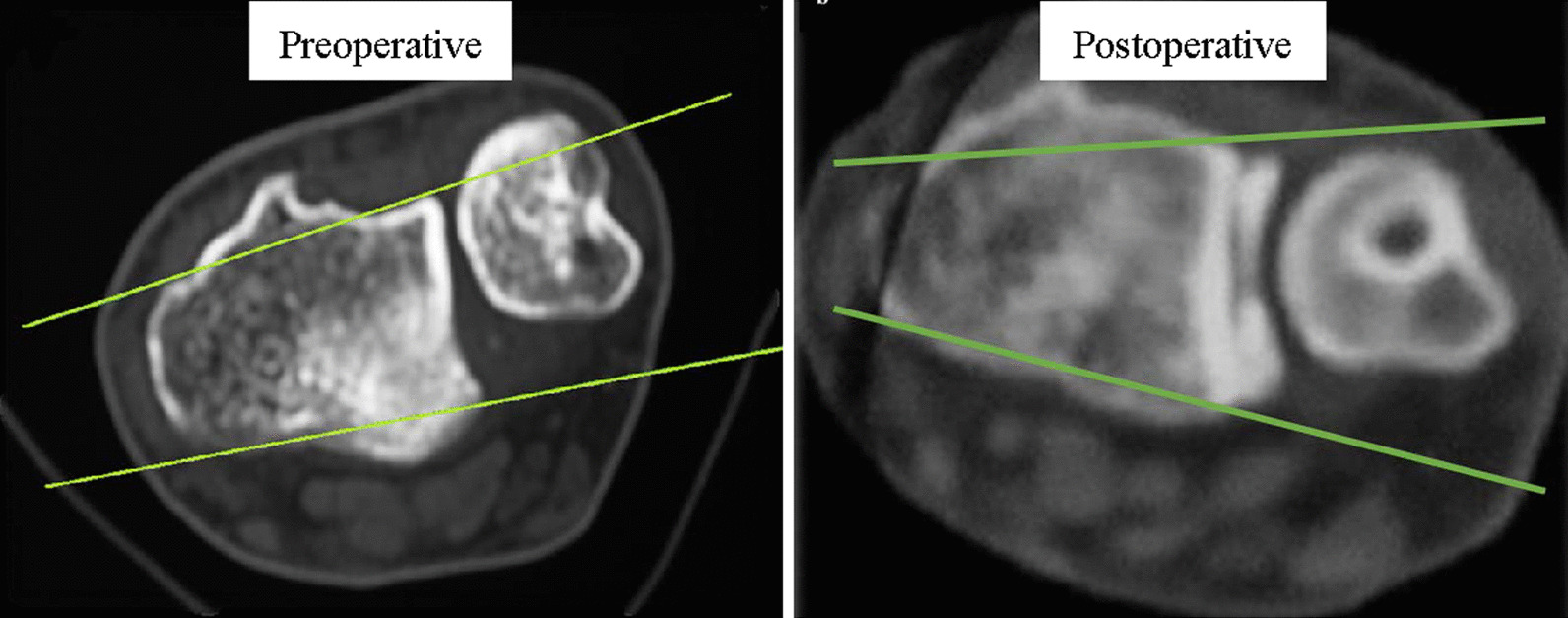
Computed tomography (CT) scan of wrist in neutral position. Preoperative CT scan shows distal radioulnar joint subluxation. Postoperative CT scan shows the ulnar head located between the two tracing lines that define the dorsal and palmar borders of the radius

Postoperative wrist CT scans revealed that the ulnar heads of all the patients were located between the two tracing lines, according to the criteria of Mino et al. [5], indicating better DRUJ positions than their positions before the surgery (Fig. 4).

## Discussion

Restoration of stability and a full, painless range of motion are the goals of treatment for chronic, posttraumatic, unstable DRUJ. When surgical management is indicated and the TFCC is irreparable, preserving the distal ulna and reconstructing both the radioulnar ligaments provide the best possibility for achieving these goals. In 1991, Hermansdorfer and Kleinman [7] first reported a series of treatments for chronic TFCC tears. Eight of 11 patients resumed painless normal activity. However, it is difficult to treat patients with chronic TFCC tears and DRUJ instability. Several TFCC reconstruction procedures have been suggested to stabilize the DRUJ [8–10]. However, most of these methods were only aimed to tighten the joint and reduce the pronation and supination capacity of the DRUJ. Hui and Linscheid [11] were the first to describe the anatomic reconstruction of dorsal DRUJ subluxation; this procedure involved TFCC reconstruction with a partial ECU tendon, and not only stabilized the DRUJ but also maintained pronation and supination. Thus, it significantly improved wrist function, and all the patients showed improved functional activity.

In 1985, Darrow et al. [12] reported the results of ulnar shortening in patients with ulnar wrist pain due to DRUJ instability, chronic TFCC tears, and Madelung deformity. They reported good or excellent results in 77% of cases. Biomechanical studies have demonstrated that shortening the ulna by just 2.0 mm significantly reduced the force transmitted to the ulna from the carpus [13, 14]. In the current study, most patients underwent ulnar shortening following TFCC reconstruction due to the presence of a positive or zero ulnar variance. Ulnar shortening can reduce TFCC loading, thus preventing further degeneration. Moreover, ulnar shortening can also tighten the TFCC, thus increasing the stability of the DRUJ and the ulnocarpal joint [15].

Thirty patients in the current study had MRI scans that indicated the presence of TFCC tears. A previous study indicated that MRI had an accuracy rate of 95% in the detection of TFCC tears [16], and therefore, MRI is highly sensitive for the diagnosis of TFCC tears. In our clinic, MRI scans revealed positive results in certain cases where arthroscopy had yielded negative findings. The current study demonstrated that the noninvasive technique is not sufficiently reliable for routine use, consistent with the findings of other studies [17–19]. Thus, arthroscopy appears to be the most accurate method for the diagnosis of TFCC tears. Several studies [20, 21] have obtained satisfactory results after the immediate repair of acute TFCC tears. For chronic cases, short-term studies have demonstrated good results for partial TFCC excision, although some long-term studies have reported a failure rate of > 30% when TFCC excision is performed without ulnar shortening [22, 23]. In 1991, Osterman and Terrill [24] reported that the debridement of the redundant cartilage remnant was highly successful, with an overall improvement rate ranging from 75 to 85%. The study demonstrated that the debridement of the degenerative TFCC and inflammatory synovium via arthroscopy could successfully alleviate symptoms. However, ulna surgery remained an option for the remaining 15–25% of patients who still exhibited symptoms.

Only three patients in this study had “fair” scores after TFCC reconstruction. Although the symptoms of all the patients improved, they still reported mild pain during work or sport, and slight limitation of wrist supination. Possible causes of this condition are the adhesion of the grafts and degenerative changes in the wrist joints. The grip strength of these patients improved to at least 65% of that of the other hand. In addition, the wrist scores were better than those before surgery. TFCC reconstruction using the free PL tendon under arthroscopy is an effective procedure. This procedure does not disturb the ECU sheath and surrounding tissues, thus preserving ulnocarpal stability.

This procedure enables intraarticular and anatomic reconstruction of both the dorsal and volar radioulnar ligaments (Fig. 4) via wrist arthroscopy. Compared to open reconstructions [25, 26], this minimally invasive procedure (not involving arthrotomy or creation of a large wound) facilitated early initiation of the rehabilitation program and shorter hospital stay. Arthroscopy also enabled clear identification of the fovea region of the distal ulnar head [27], which allowed precise creation of the tunnel using a microvector drill guide. The tendon graft was passed from the dorsal and volar sides of the distal radius to the fovea region of the distal ulnar head, which was in close proximity to the rotation center of the ulnar long axis.

**Fig. 5:**
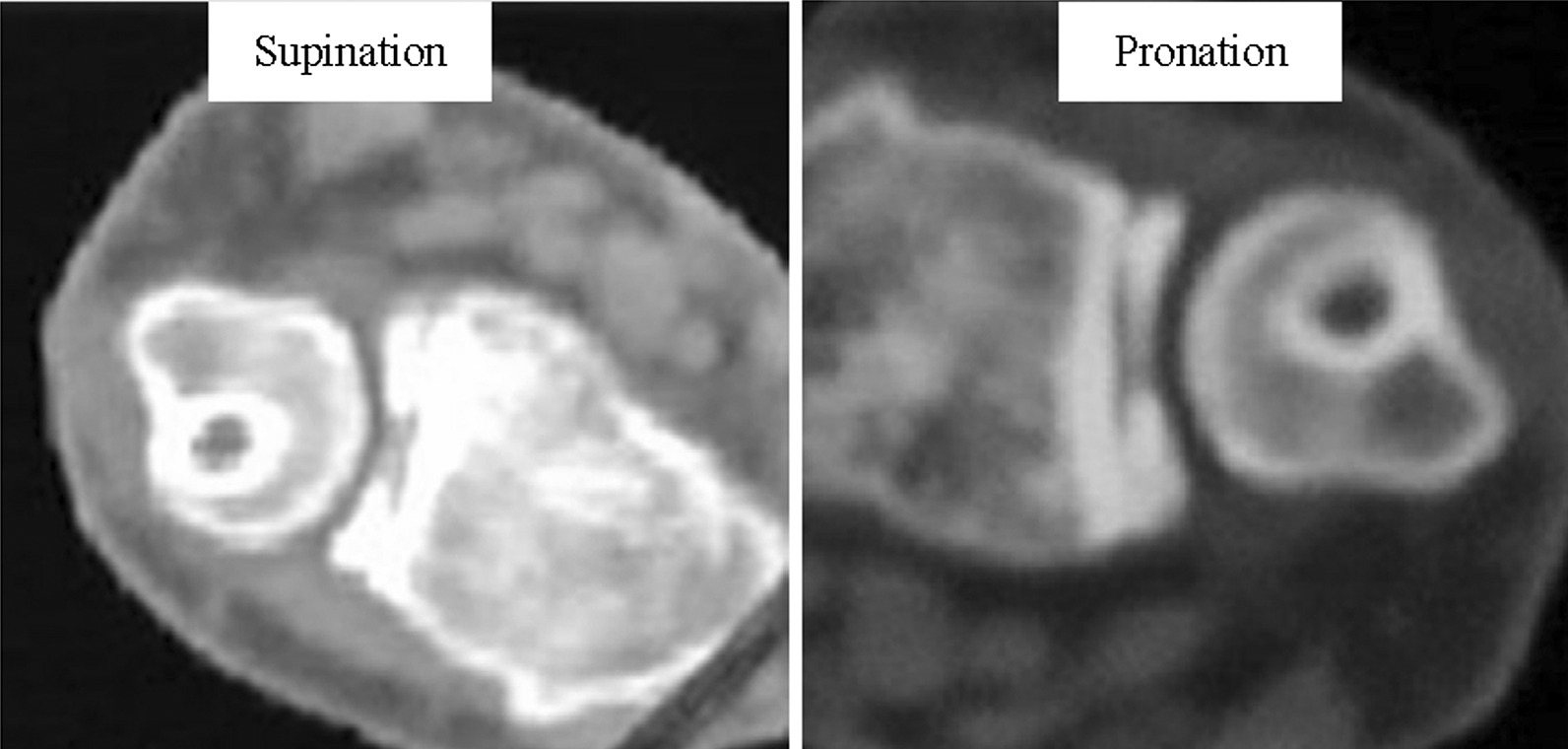
Postoperative computed tomography of wrist in supine and prone position. The supine and prone positions of the wrist demonstrate good distal radioulnar joint congruency and no motion limitation

In this study, the tendon graft was able to prevent DRUJ diastasis and subluxation without impairing the range of motion (Fig. 5). After the tendon graft was removed from the ulnar tunnel, we used an anchor sutured to fix tendon graft to ulna and suture with the surrounding tissue via a small incision over the ulnar neck without extensive dissection of the soft tissue, including the dorsal DRUJ capsule and extensor retinaculum. We also did not loop the tendon graft limbs around the ulnar neck [25] because this more invasive method has not been proven to improve graft tension or DRUJ stability. We used palmaris longus for reconstruction of irreparable TFCC. Tendon had more flexibility than ligament, so that it would be looser under postoperative rehabilitation than its original reconstruction status. So, we will control the tension as high as possible during the operation. All of the patients were satisfied to their wrist ROM and the pronation and supination functions after the adequate rehabilitation for several months. This repair method seemed to be effective from the restoration of the patients’ function and greatly biomechanically improvement for them in their daily functions [28].

For more chronic cases with preexisting DRUJ arthritis, the current procedure is contraindicated, and other techniques such as the Darrach and Sauvé-Kapandji procedures are recommended for pain relief. However, the results of the Darrach procedure are generally poor in patients with greater activity demands [29, 30]. The Sauvé-Kapandji procedure yielded good results for patients who were young or those with greater activity demands [31]. However, the potential complications included nonunion or delayed union of the arthrodesis, fibrous or osseous union at the pseudoarthrosis, and painful instability at the proximal ulna stump [32]. In the current study, the patients were relatively young (age range, 19–34 years; mean age, 26.4 years) and the period from the event causing the tear to surgery was short (range, 14–28 months; mean, 22.6 months). All patients underwent early TFCC reconstruction prior to degeneration. At the postoperative follow-up examination, 56.7% of patients achieved “excellent” results and 34.3% of patients had “good” results. We suggest that surgery should be performed at an early stage, immediately once reconstruction is indicated, and before the development of degenerative changes secondary to DRUJ instability or ulnocarpal impaction.

This procedure establishes a potentially satisfactory head-notch relationship, restores TFCC integrity and stability, maintains DRUJ supination and pronation motion, and reduces the force transmitted to the ulna. This would consequently reduce symptoms, improve wrist function, and enable patients to perform work, sport, and military training activities.

## Conclusions

From our study, we found that TFCC reconstruction with the free PL tendon under arthroscopy seemed to be a good minimal invasive surgical method for treating the patients diagnosed as irreparable TFCC injury with chronic posttraumatic DRUJ instability.

## Data Availability

All data generated or analyzed during this study are included in this published article.

## Abbreviations

CT: Computed tomography
DRUJ: Distal radioulnar joint
ECU: Extensor carpi ulnaris
MRI: Magnetic resonance
NSAIDs: Nonsteroidal anti-inflammatory drugs
PL: Palmaris longus
TFCC: Triangular fibrocartilage complex
